# Laparoscopic cholecystectomy for cholecystolithiasis with Dubin–Johnson syndrome

**DOI:** 10.1002/jgh3.12204

**Published:** 2019-07-22

**Authors:** Baolin Wang, Shouda Yang, Xiaoming Hu, Yongfeng Zhang

**Affiliations:** ^1^ Centre of General Surgery, Hospital of People's Liberation Army Urumqi City Xinjiang China

**Keywords:** cholecystolithiasis, Dubin–Johnson syndrome, laparoscopic cholecystectomy

## Abstract

Dubin–Johnson syndrome is a rare, autosomal recessive hereditary disease, commonly known as “black liver disease.” It is of great interest for surgeons to accidentally find the “black liver” during laparoscopic cholecystectomy (LC). We performed an emergency LC for cholecystolithiasis with Dubin–Johnson syndrome in 2013. We only performed cholecystectomy and liver tissue biopsy. Following a 5‐year follow‐up period, the patient does not appear to have abdominal pain and any other discomfort. Dubin–Johnson syndrome has no significant relationship with the occurrence of cholecystolithiasis and generally requires no special treatment. It is necessary to avoid misdiagnosis and overtreatment in the clinic.

## Graphical abstract

Dubin–Johnson syndrome is a rare bilirubin metabolism disorder, which does not increase the incidence of cholecystolithiasis. There is generally no need for special treatment, but it is necessary to avoid misdiagnosis and overtreatment in the clinic.

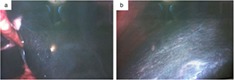

## Introduction

Dubin‐Johnson syndrome is an extremely rare disease and case reports are distributed around the world. However, there are few cases of the “black liver” during laparoscopic cholecystectomy (LC). It will bring great psychological pressure to the surgeon if it appears in laparoscopic surgery. How to choose the surgical method and intraoperative treatment is extremely important for postoperative recovery. Here the case report can be used as a reference.

## Case Report

A 36‐year‐old male patient presented with upper abdominal pain. Physical examination demonstrated acute illness, increased heart rate, slightly yellow bilateral sclera, pain in the right upper abdominal, rebound tenderness, and positive Murphy's sign. Abdominal computed tomography (CT) identified cholecystolithiasis and enlarged gallbladder and no expansion of intrahepatic bile duct. Laboratory tests demonstrated a white blood cell count of 13.6 × 10^9^/L, total bilirubin of 64 μmol/L, conjugated bilirubin of 47 μmol/L, and positive urine bilirubin. The patient denied history of hepatitis, typhoid fever, tuberculosis and other infectious diseases, blood transfusion, and exposure to toxins. The evidence suggested a diagnosis of cholecystolithiasis. Emergency laparoscopic cholecystectomy (LC) was carried out.

**Figure 1:**
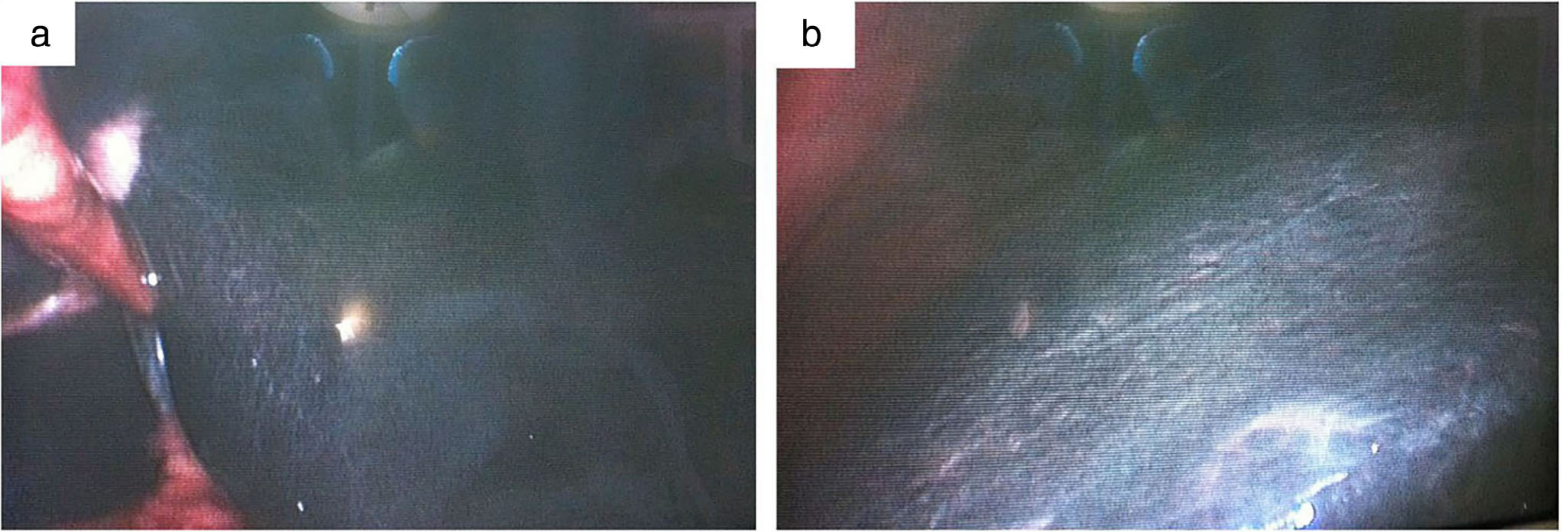
The left (a) and right (b) livers were blackish brown, and the liver surface was smooth and nodular under laparoscopy.

**Figure 2:**
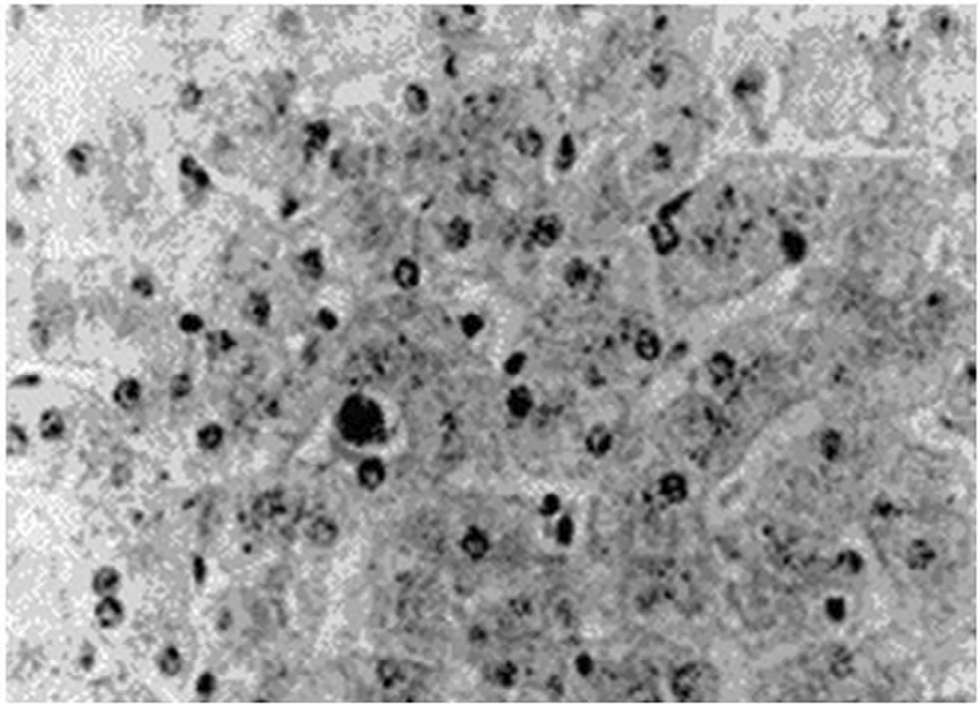
Liver biopsy showed mild turbidity of liver cells and a large amount of brownish particles deposited in the cytoplasm (HE × 400).

We observed a blackish brown liver (Fig. 1), gallbladder congestion, and swelling of approximately 15 × 10 × 4 cm in size under laparoscopy. Considering that there was no expansion in the common bile duct, we only performed cholecystectomy and hepatic lesion biopsy. Biopsy of the hepatic lesion demonstrated Dubin–Johnson syndrome (Fig. 2). Following a 5‐year follow‐up period with the patient, conjugated bilirubin has been stable at around 50 μmol/L. The patient has had no recurrence of cholecystolithiasis, no pain in the liver area, and no adverse effect on the quality of life.

## Discussion

Dubin–Johnson syndrome is a rare hereditary disease, commonly known as “black liver disease.” It is caused by the mutation of the human MRP2 gene and is linked to bilirubin export disorder. Sibship has a higher prevalence, which is homozygous of the pathogenic gene. Cases have been reported around the world. It mainly manifests as long‐term fluctuation jaundice. However, biopsy of the liver tissue is required for the diagnosis.

Dubin–Johnson syndrome may be misdiagnosed as obstruction and cholestasis if a blackish brown liver is observed during LC. Once a blindly expanded operation, bile duct exploration, and T‐tube drainage have been performed for biliary obstruction and cholestasis, it will increase the patient's pain.

Dubin–Johnson syndrome may be overtreated due to chronic unstable jaundice. Through 5 years of follow‐up, it was found that this syndrome does not increase the incidence of cholecystolithiasis. It generally does not require special treatment. It is necessary to avoid misdiagnosis and overtreatment in clinic.

